# *Nypa fruticans* Wurmb Extract Recovered Compromised Immune Status Induced by Forced Swimming in a Mouse Model

**DOI:** 10.4014/jmb.2411.11006

**Published:** 2024-12-12

**Authors:** Gun-Dong Kim, Sang Hyuk Yoo, Ju Hye Song, Kyung min Lim, Eun Yeong Lim, Ji Yeon Yoo, Da-Kyoung Lee, Yong-Baik Cho, Heong-Jun Yu, So-Young Lee, Hee Soon Shin

**Affiliations:** 1 Food Functionality Research Division, Korea Food Research Institute (KFRI), Wanju 55365, Republic of Korea; 2 Department of Food Biotechnology, Korea University of Science and Technology (UST), Daejeon 34113, Republic of Korea; 3 Department of Food Science and Technology, Jeonbuk National University, Jeonju 54896, Republic of Korea; 4 K Pharms, Incheon 21695, Republic of Korea

**Keywords:** *Nypa fruticans* Wurmb, forced swimming, immune compromised model, natural killer cell

## Abstract

*Nypa fruticans* Wurmb is known to contain large amounts of polyphenols and flavonoids with antioxidative and anti-inflammatory effects. However, the biological and physiological functions of *N. fruticans* have not been scientifically investigated. Thus, we investigated the immunomodulatory effect of *N. fruticans* hot water extract (YSK-N) in mice using an immune compromised model established by forced swimming (FS). Intensive exercise decreased body weight, organ index, and various immunological parameters in FS mice. However, oral administration of YSK-N significantly restored the FS-induced decreases in body, thymus, and spleen weights, as well as the reduction in the numbers of white blood cells and lymphocytes in the whole blood of mice. Additionally, YSK-N increased splenic cell proliferation in the absence and presence of concanavalin A or lipopolysaccharide stimulation in a concentration-dependent manner. Notably, YSK-N enhanced the cytotoxic activity of natural killer cells against YAC-1 tumor cells under immunosuppressive conditions. Furthermore, YSK-N supplementation reverted the FS-induced downregulation in immunoglobulin production and *Il2*, *Il6*, *Il12*, *Ifnγ*, *Gzmb*, and *Prf1* mRNA expression. Therefore, our observations suggested that YSK-N promotes immune function and has potential as an immunomodulatory agent.

## Introduction

The human body maintains immune homeostasis through a complex and effective immune system composed of a network of cells, tissues, and organs that protects it against infections and diseases caused by various external antigens and pathogens, including bacteria, viruses, fungi, and harmful substances [1]. Immune cells that are produced in the bone marrow and matured in the thymus, spleen, and lymph nodes mediate immune responses [2]. Innate immune cells, such as neutrophils, monocytes, macrophages, dendritic cells, and natural killer (NK) cells, contribute to the onset of immune response and facilitate the activation of adaptive immune responses through antigen presentation [3]. The adaptive immune response, which is highly specific to pathogens, encompasses two major categories including cell-mediated immune response driven by T lymphocytes and humoral immune response mediated by B lymphocytes [4, 5]. T lymphocyte populations include cytotoxic T cells, killer T cells, and CD8 T cells, which specifically bind to virus-infected host and cancer cells to destroy target cells, as well as helper T cells, which regulate the activation of other immune cells and mediate immune responses [6]. T lymphocytes produce cytokines, including interleukin (IL) 2, IL6, IL12, and interferon γ, which are recognized as immunostimulators and regulators of immune responses [7]. B lymphocytes comprise approximately 15% of the entire lymphocyte population and produce antibodies specific to foreign antigens [8].

When immune regulatory functions are compromised by various environmental factors, it can lead to T lymphocyte imbalance and unbridled immune response, thereby decreasing immune homeostasis and contributing to various diseases driven by chronic or autoimmune responses [9]. Additionally, overtraining syndrome, induced by irregular or intensive exercise, has been reported to diminish immune function by reducing neutrophil activity, lowering immunoglobulin production, suppressing lymphocyte proliferation, and decreasing the number and function of NK cells [10-12]. As a reduction in immobility time indicates improved stamina and endurance for sustaining swimming, antidepressant, anti-fatigue, endurance, and physical fitness effects are assessed using the forced swimming (FS) mouse experimental model [13, 14]. As FS induces significant psychophysiological stressors associated with behavioral, physiological, endocrine, and immune function changes, which lead to alterations in levels of various immune-related markers, this model is an effective assessment tool for screening immunomodulatory agents [15-17].

*Nypa fruticans* Wurmb belongs to the *Araceae* family and is grown in India, Malaysia, Indonesia, and the Philippines [18]. *N. fruticans* is traditionally used as a pain reliever, sedative, and treatment agent for leprosy, tuberculosis, and sore throat [19]. Additionally, *N. fruticans* contains large amounts of polyphenolic and flavonoid constituents, such as chlorogenic acid, protocatechuic acid, and kaempferol, which have antioxidant and anti-inflammatory properties [19]. However, there exists an underutilization of bioactive functions of *N. fruticans*, with scope for scientific investigation on the immunomodulatory activity as a previously unevaluated aspect. Therefore, in this study, the immune regulatory effects of *N. fruticans* were investigated in an FS-induced immune compromised mouse model.

## Materials and Methods

### Plant Material and YSK-N Preparation

*N. fruticans* Wurmb (2 kg), obtained from Golden Hands Co., Ltd. (Republic of Korea) was rinsed with distilled water (DW), followed by reflux extraction at 95 to 100°C for 5 h with the addition of 20 L of DW. After filtering the first extract, a second reflux extraction was conducted on the remaining *N. fruticans* with the addition of 10 times the volume of DW for 5 h at 95 to 100°C. The first and second extracts were combined, concentrated at 60°C to achieve a concentration of 10-15 Brix (optimized for freeze drying), subsequently freeze-dried (YSK-N), and then stored at −80°C until use.

### Cell Preparation from Spleen and Draining and Mesenteric Lymph Nodes of Mice

Five-week-old male BALB/c mice were euthanized according to the guidelines of the Institutional Animal Care and Use Committee of the Korea Food Research Institute (approval number: KFRI-M-24050). The harvested axillary, branchial, inguinal, and mesenteric lymph nodes, and spleen were placed in RPMI-1640 medium containing 10% FBS and 1% penicillin-streptomycin with collagenase D digestion medium. Using a plunger of a 3 ml syringe to homogenize the lymph nodes and spleen, cell suspensions were filtered by passing through a 40 μm cell strainer, and centrifuged at 1,500 rpm at 4°C for 5 min. After washing, cells were seeded into 24-well cell culture plates at a density of 1 × 10^6^ cells/well, cultured in a humidified atmosphere of 5% CO_2_ and 95% air at 37°C, and subsequently used in *ex vivo* experiments.

### Cell Viability and Proliferation Assay

To assess the cytotoxic and proliferative effects of YSK-N on immune cells from draining and mesenteric lymph nodes, a water-soluble tetrazolium 1 (WST-1) assay was performed. Splenocytes were cultured in 48-well plates at a density of 2.5 to 5 × 10^6^ cells/well and treated with 25 μg/ml YSK-N in the absence or presence of 1 μg/ml concanavalin A (ConA). The cells were then incubated at 37°C for 48 h in a humidified atmosphere composed of 5% CO_2_ and 95% air. After incubation, the cells were centrifuged at 1,500 rpm for 5 min to allow them to adhere to the bottom of the plate, and the WST-1 solution was mixed with the medium at a ratio of 1:10 in each well. Subsequently, cells were incubated at 37°C for 30 min, and the absorbance of each well was measured at 450 nm using an Epoch microplate reader (BioTek, USA).

### Measurement of Cytokine Level

Draining and mesenteric lymph node cells were obtained from mice, cultured in 24-well culture plates at a density of 1 × 10^6^ cells/wells, treated with 25 μg/ml YSK-N and 1 μg/ml ConA, and incubated at 37°C for 48 h in a humidified 5% CO_2_ incubator. Culture supernatants were quantified, using a Bio-Rad protein assay kit based on the Bradford protein assay (Bio-Rad, USA), and subsequently used for ELISA. IL2, IL6, and IL12p40 levels were measured using an ELISA kit (BD Biosciences, USA) according to the manufacturer's instructions.

### Animal Study

All animal procedures were performed according to the Institutional Animal Care and Use Committee guidelines of the Korea Food Research Institute (approval number: KFRI-M-24050). Four-week-old male BALB/C mice were obtained from Orient Bio Inc. (Republic of Korea), acclimated for 1 week under specific pathogen-free conditions in a consistent environment, observing a regular 12 h light/dark cycle, with the temperature and relative humidity maintained at 23 ± 2°C and 50 ± 5%, respectively, and randomly divided into five groups (*n* = 5): negative control, forced swimming (FS), 200 mg/kg red ginseng (RG)-treated (positive control), 200 mg/kg YSK-N-treated, and 400 mg/kg YSK-N-treated groups. To establish an immune compromised model, FS was modified utilizing previous studies [10, 14, 20]. Briefly, the mice were conducted daily for 15 days, involving a swimming session in a container of water with a pump-installed flow rate of 10 L/min for 15 min, followed by a rest for 5 min, and a second swimming session for 15 min. This setup ensured FS because the forced water current in the container prevented the mice from floating. From day 1 to day 21, daily treatments were administered orally with the negative control group receiving 200 μl PBS, while YSK-N groups received 200 mg/kg or 400 mg/kg YSK-N resuspended in 200 μl PBS. RG (ginsenosides Rg_1_, Rb_1_, and Rg_3_ contents was 16.5 mg/g) was provided by Huons Foodience Co., Ltd. (Republic of Korea), and at a final concentration of 200 mg/kg was administered daily from days 1 to 21 as a positive control. The mice were euthanized on day 21 and their blood and organs were collected for further study. Blood samples from each mouse were analyzed for the hematopoietic compartment and the numbers of lymphocytes and white blood cells using an automatic blood cell analyzer (Hemavet 950 FS; Drew Scientific Inc., USA).

### Assessment of Splenic Natural Kller Cell Cytotoxicity

Splenocytes from mice were co-cultured with 4 × 10^4^ cells/well of YAC-1 cells (ATCC, USA) for 4 h in an incubator at 37°C. Flow cytometry-based cytotoxicity assays were performed using activated splenocytes as effectors and CellTrace Violet (CTV)-stained YAC-1 cells as natural killer (NK) cell-targeted tumor cells, with an effector:target (E:T) ratio of 20:1. The cytotoxicity of splenic NK cells towards YAC-1 cells was also analyzed using CytoFLEX (Beckman Coulter, Inc., USA). The results were interpreted using CytExpert software (Beckman Coulter, Inc.) and presented as the mean percentage of CTV^+^ 7-Aminoactinomycin D^+^ YAC-1 cells.

### RNA Extraction and Real-Time Quantitative Polymerase Chain Reaction (RT-qPCR)

To extract mRNA from the spleen tissues, QIAzol Lysis Reagent was added to the individual spleen samples collected from each group of mice. The tissues were homogenized four times for 20 s at 20 Hz, using a TissueLyser II (Qiagen, USA), and then extracted using a High Pure RNA Tissue Kit (Roche, Germany), according to the manufacturer's instructions. Total RNA (1 μg) was reverse transcribed with ReverTra Ace qPCR RT Master Mix (Toyobo, Japan) using a Bio-Rad C1000 Thermal Cycler (USA). RT-qPCR was performed using SYBR Green PCR Master Mix (Toyobo) on an Applied Biosystems Step One Plus real-time PCR system (USA) in the presence of gene-specific primers. The sequences of the primers used were as follows: *Il2* forward, 5'-GCGGCATGTTCT GGATTTGACTC-3' and reverse, 5'-CCACCACAGTTGCTGACTCATC-3'; *Il6* forward, 5'-TAGTCCTTCCTA CCCCAATTTCC-3' and reverse, 5'-TTGGTCCTTAGCCACTCCTTC-3'; *Il12* forward, 5'-ACGAGAGTTGCC TGGCTACTAG-3' and reverse, 5'-CCTCATAGATGCTACCAAGGCAC-3'; *Ifnγ* forward, 5'-CAGCAACAG CAAGGCGAAAAAGG-3' and reverse, 5'-TTTCCGCTTCCTGAGGCTGGAT-3'; *Gzmb* forward, 5'-CAGGAG AAGACCCAGCAAGTCA-3' and reverse, 5'-CTCACAGCTCTAGTCCTCTTGG-3'; *Prf1* forward, 5'-ACA CAGTAGAGTGTCGCATGTAC-3' and reverse, 5'-GTGGAGCTGTTAAAGTTGCGGG-3'; *Rplp0*, used as internal control gene: forward, 5'-GCTCCAAGCAGATGCAGCA-3' and reverse, 5'-CCGGATGTGAGGCAG CAG-3'.

### Quantification of Immunoglobulin and Corticosterone Levels

Serum levels of immunoglobulin (Ig) G, IgA, secretory IgA, and corticosterone were measured using commercially available ELISA kits (BD Biosciences, USA) according to the manufacturer’s instructions. Briefly, each well was coated overnight with a capture antibody. Next, each well was washed with a washing buffer and blocked by adding the assay solution for 1 h. Standards and supernatants were diluted with the assay solution and added to the appropriate wells for 2 h. The detection antibody and horseradish peroxidase were mixed with reagent diluents and added to appropriate wells for 1 h. The substrate solution was then added to the appropriate wells for 30 min. After 30 min, a stop solution was added to each well. The absorbance was measured at 450 nm using an Epoch microplate reader (BioTek, USA).

### Statistical Analyses

All data were analyzed using GraphPad Prism version 10.3.1 (GraphPad Software, USA). The statistical significance of differences between two groups was analyzed using Student’s *t*-test, and multiple groups were statistically compared using one-way analysis of variance (ANOVA) followed by a least significant difference test. All data were expressed as mean ± standard deviation. A *p*-value < 0.05 was considered to be statistically significant (*p < 0.05, **p < 0.01, and ***p < 0.001).

## Results

### YSK-N Increased Immune Response-Associated Cytokine Production and Splenic NK Cell Cytotoxicity in *ex vivo* Experiments

**Fig. 1:**
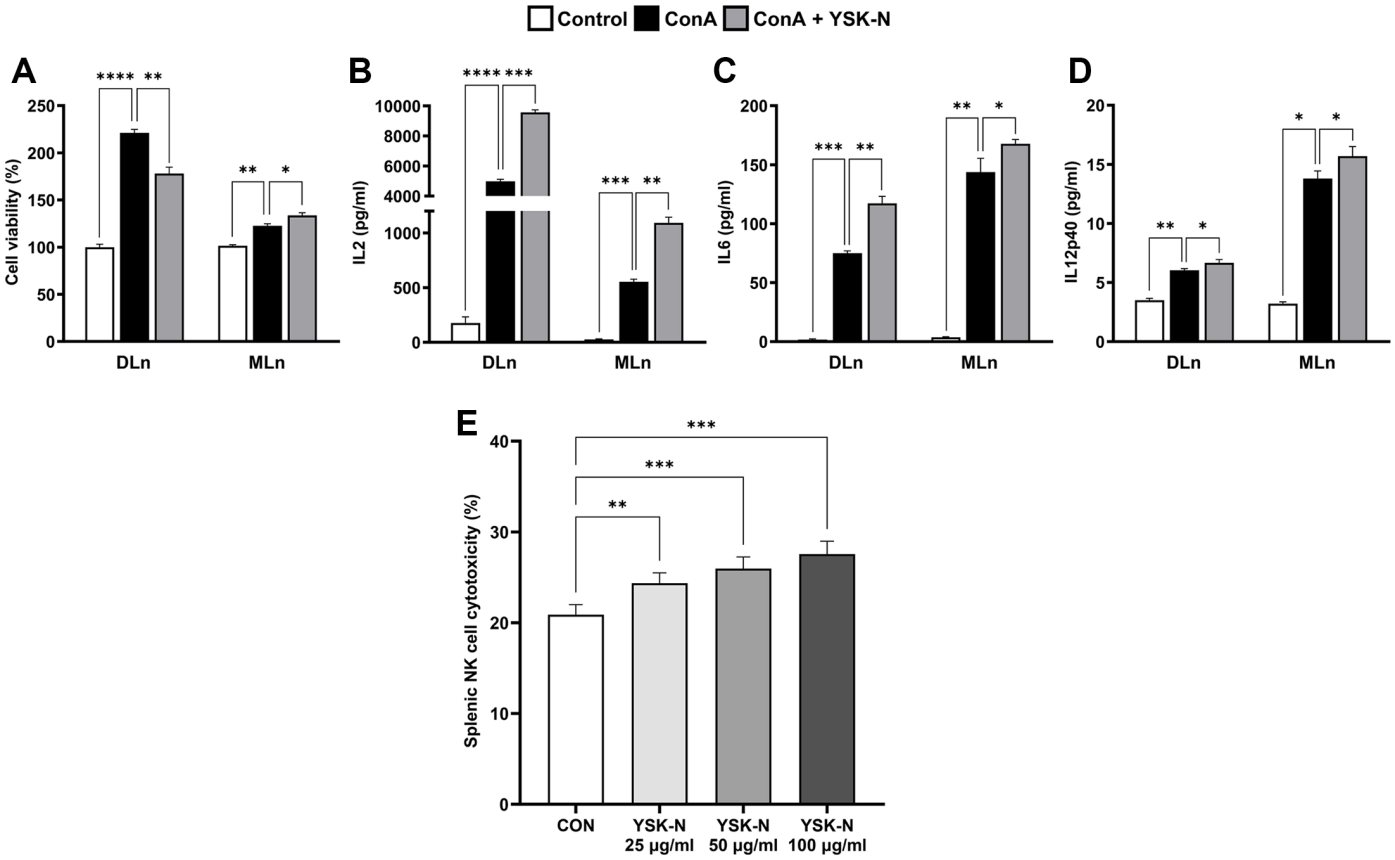
Effect of YSK-N on the proliferation and function of cells derived from draining and mesenteric lymph nodes, and splenic NK cell cytotoxicity. (**A**) ConA (1 μg/ml)-stimulated cell treated with 25 μg/ml YSK-N and viability was assessed using the WST-1 assay (*n* = 3). (**B-D**) The levels of IL2 (**B**) IL6 (**C**) and IL12p40 (**D**) were measured using ELISA (*n* = 3). (**E**) Splenocytes (8 × 10^5^ cells/well) from mice were treated with 25, 50, and 100 μg/ml YSK-N for 24 h. After 24 h, splenocytes were co-cultured with 2 × 10^5^ cells/well of CTV-stained YAC-1 cells for 4 h in an incubator at 37°C. The cytotoxicity of splenic NK cells towards YAC-1 cells was analyzed using CytoFLEX flow cytometry (Beckman Coulter, Inc.). Data were analyzed using Student’s *t*-test as mean ± SD values (*p < 0.05, **p < 0.01, and ***p < 0.001). ConA, concanavalin A; DLn, draining lymph node; MLn, mesenteric lymph node.

IL2, IL6, and IL12 are immunostimulatory factors that are induced by infections or tissue damage, initiating hematopoiesis and innate immune responses such as host defense and promoting T lymphocyte proliferation and activation [21-23]. These cytokines play crucial roles in the development of adaptive immunity and various immune responses [24]. Additionally, NK cells play a pivotal role in innate immunity, such as infected cells and tumor cell elimination [25]. Therefore, promoting the NK cell cytotoxicity is regarded as an enhancement of immune function. To identify the immunomodulatory functions of YSK-N, spleen and draining and mesenteric lymph nodes of mice, which are widely used in studies on immune function, were utilized in *ex vivo* experiments (Fig. 1). As shown in Fig. 1A, YSK-N promoted cell proliferation without cytotoxicity in cells isolated from both the draining and mesenteric lymph nodes of mice (Fig. 1A). Additionally, YSK-N treatment significantly increased IL2, IL6, and IL12p40 production in lymphocytes isolated from these draining and mesenteric lymph nodes following ConA stimulation that used as activate T lymphocytes and immune response [26] (Fig. 1B-1D). Next, we investigated whether YSK-N affects splenic NK cell cytotoxicity against YAC-1 cells. As shown in Fig. 1E, treatment with YSK-N at concentrations of 25, 50, and 100 μg/ml significantly increased splenic NK cell cytotoxicity (24.4%, 26.0%, 27.6%) against YAC-1 cells compared to that in the control (20.9%) (Fig. 1E). Collectively, our observations demonstrated that YSK-N enhanced the proliferation and function of lymphocytes and cytotoxicity of splenic NK cells.

### YSK-N Restored Body Weight and Organ Index in FS-Induced Immune Compromised Model

**Fig. 2:**
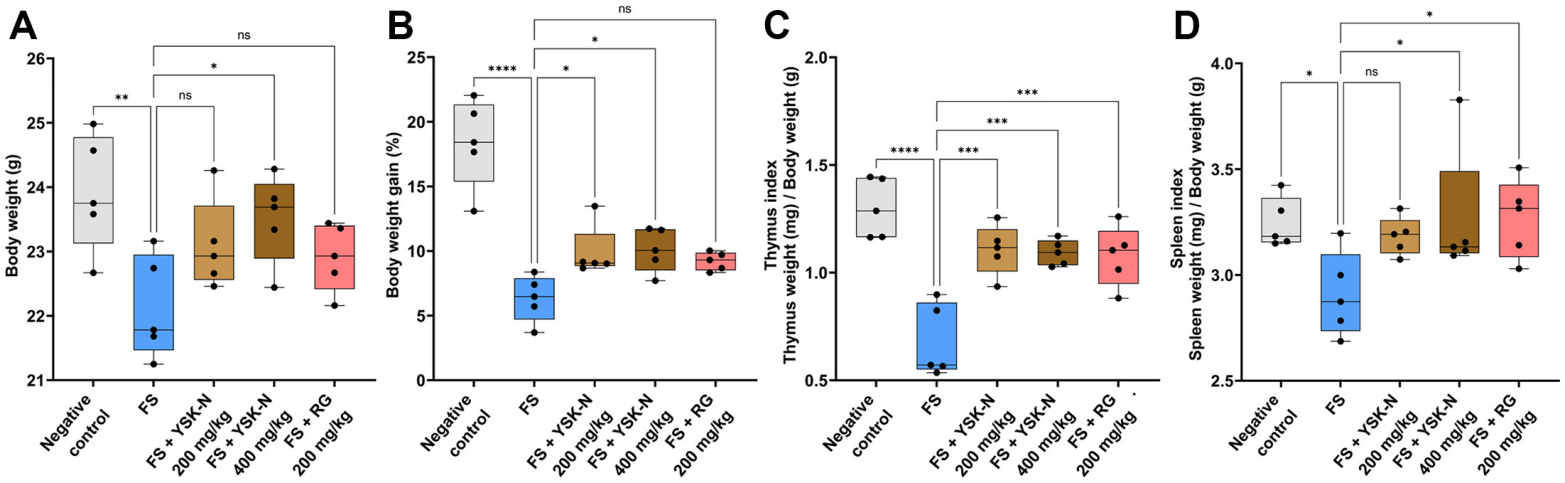
Effect of YSK-N on the weights of body and immune organs in the FS model. (**A**) On day 21, mice were anesthetized with isoflurane, and individual body weight was measured (*n* = 5). (**B**) Weight gain was assessed using the following formula: final body weight (g) – initial body weight (g) (*n* = 5). (C and D) Thymus (**C**) and spleen (**D**) index were evaluated using the following formula: organ weight (mg) / body weight (g) (*n* = 5). Data were presented as mean ± SD and analyzed using one-way analysis of variance (**ANOVA**) (*p < 0.05, **p < 0.01, and ***p < 0.001). FS, forced swimming; YSK-N, *Nypa fruticans* Wurmb hot water extract; RG, red ginseng.

To investigate the immunomodulatory efficacy of YSK-N in an FS-induced immunosuppressed mouse model, YSK-N was orally administered for three weeks at final concentrations of 200 and 400 mg/kg. As shown in Fig. 2A and 2B, body weight and body weight gain were significantly lower in the FS group than in the negative control group on day 21 (Fig. 2A and 2B). However, YSK-N supplementation reversed the FS-induced decrease in body weight in a concentration-dependent manner (Fig. 2A and 2B). Bone marrow-derived T lymphocytes undergo differentiation, selection, and maturation in the thymus, a primary immune organ and immune responses to blood-derived antigens and activation of immune cells occur in the spleen, a secondary immune organ [27, 28]. These organs play major roles in immunity and immune homeostasis, and changes in organ weight are used as major immune indicators [29, 30]. Accordingly, we assessed the effect of YSK-N on the thymus and spleen index, which was defined as the ratio of the weight of these immune organs to the body weight of individual mice. FS induced a significant decrease in thymus and spleen weights compared to those in the negative control group (Fig. 2C and 2D). However, the administration of YSK-N recovered the FS-induced decrease in the immune organ index (Fig. 2C and 2D). Taken together, our results showed that YSK-N reversed the FS-induced decreases in body, thymus, and spleen weights in a mouse model.

### Effect of YSK-N on Hematological Parameters of the FS Model

**Fig. 3:**
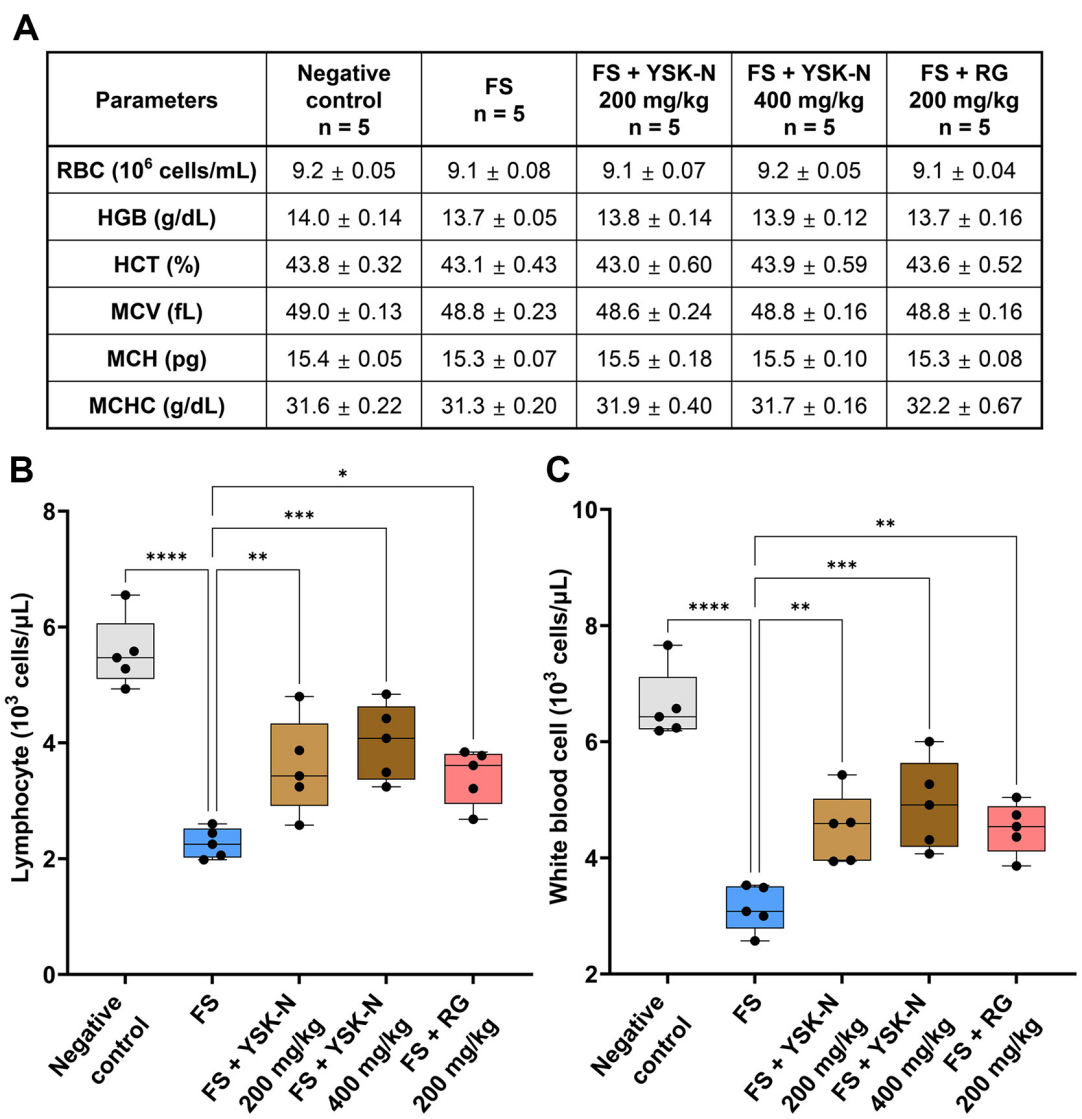
Effect of YSK-N on hematological parameters in the FS model. (**A-C**) Total blood of mice was individually analyzed with a Hemavet 950 FS hematology profiling unit (*n* = 5). Table (**A**) and graphs (B and C) represent the mean numbers of lymphocytes (**B**) and white blood cells (**C**) in the whole blood with standard deviations (*n* = 5). Data were analyzed using one-way analysis of variance (**ANOVA**). All values have been reported as mean ± SD. (*p < 0.05, **p < 0.01, and ***p < 0.001). FS, forced swimming; YSK-N, *Nypa fruticans* Wurmb hot water extract; RG, red ginseng.

Next, we examined whether YSK-N affected the mouse hematopoietic cell compartment. As shown in Fig. 3A, the hematological components revealed no significant differences in red blood cell counts, hemoglobin concentration, hematocrit, mean corpuscular volume, mean corpuscular hemoglobin, and mean corpuscular hemoglobin concentration under FS conditions in the presence or absence of YSK-N supplementation (Fig. 3A). Further analysis of the lymphocyte and white blood cell populations in whole blood revealed a significant decrease in cell number in the FS group compared to that in the negative control group (Fig. 3B and 3C). However, oral administration of YSK-N recovered the numbers of lymphocytes and white blood cells to those of the negative control group in a concentration-dependent manner (Fig. 3B and 3C). Collectively, our results showed that YSK-N recovered the decreased population of immune cells, including lymphocytes and white blood cells, in the whole blood of mice under FS conditions.

### YSK-N Increased Lymphocyte Proliferation and NK Cell Function among Splenocytes from FS-Induced Immune Compromised Model

**Fig. 4:**
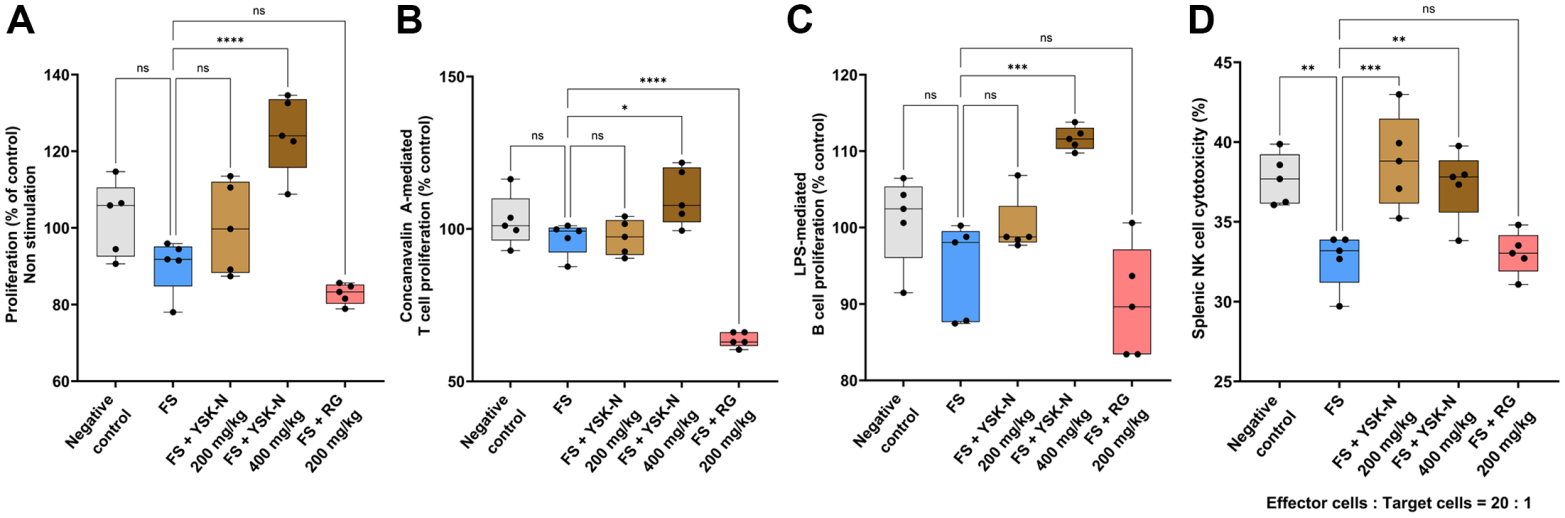
Effect of YSK-N on lymphocyte proliferation and NK cell activity in the FS model. (**A-C**) On day 21, mice were anesthetized with isoflurane, their spleens were excised, and splenocytes were isolated (*n* = 5). Splenocyte viability (**A**) was assessed in the presence of either 1 μg/ml of ConA (**B**) or 1 μg/ml of LPS (**C**) using the WST-1 assay (*n* = 5). (**C**) Splenic NK cell cytotoxicity against YAC-1 tumor cells was analyzed using the CytoFLEX flow cytometry system and represented as the mean percentage of CellTrace Violet^+^ 7-Aminoactinomycin D^+^ YAC-1 cells (*n* = 5). Data were presented as mean ± SD and analyzed using one-way analysis of variance (**ANOVA**) (*p < 0.05, **p < 0.01, and ***p < 0.001). FS, forced swimming; YSK-N, *Nypa fruticans* Wurmb hot water extract; RG, red ginseng.

As shown in Fig. 1, YSK-N enhanced the proliferation and function of lymphocytes isolated from the draining and mesenteric lymph nodes of mice following ConA stimulation in an *ex vivo* experiment (Fig. 1). Therefore, we investigated whether YSK-N regulated splenocyte proliferation in an FS-induced immune compromised mouse model. As anticipated, YSK-N restored the FS-induced decrease in splenocyte proliferation (Fig. 4A). Notably, 400 mg/kg YSK-N supplementation significantly promoted splenocyte proliferation compared to the FS group, which was even higher than that in the negative control group (Fig. 4A). Accordingly, we assessed the effects of YSK-N on the proliferation of T and B lymphocytes in response to specific stimuli, such as ConA and lipopolysaccharide (LPS). T lymphocyte proliferation in splenocytes after ConA stimulation was lower in the FS group than in the negative control group (Fig. 4B). However, YSK-N supplementation reversed this decrease in T lymphocyte proliferation in a concentration-dependent manner (Fig. 4B). Consistent with these observations, B lymphocyte proliferation in splenocytes after LPS stimulation was lower in the FS group than in the negative control group (Fig. 4C). Interestingly, oral administration of YSK-N elevated B lymphocyte proliferation in a concentration-dependent manner compared to that in the FS group (Fig. 4C). NK cells are major components of innate immunity and are responsible for responding to pathogens, such as bacteria and viruses, in addition to tumor cells [25]. In mice, approximately 2-3 million is the largest number of NK cells reported to be present in the spleen [31]. Recent studies have demonstrated that NK cells are responsive to exercises, such as running and swimming, and intense or prolonged physical exertion may reduce the number and hinder the function of circulating NK cells [32-34]. Therefore, we investigated whether YSK-N affects splenic NK cell cytotoxicity in an FS-induced immune compromised mouse model. FS significantly impaired splenic NK cell cytotoxicity (32.7%) against YAC-1 cells compared to that in the negative control group (37.7%), at an E:T ratio of 20:1 (Fig. 4D). However, YSK-N supplementation at 200 mg/kg (38.8%) and 400 mg/kg (37.3%) significantly elevated NK cell cytotoxicity compared to the FS group (Fig. 4D). Taken together, our observations showed that YSK-N enhances both the proliferation of splenocytes, including T and B lymphocytes, and the splenic NK cell cytotoxicity.

### Effect of YSK-N on Immunoglobulin and Corticosterone Production in FS Mouse Model

**Fig. 5:**
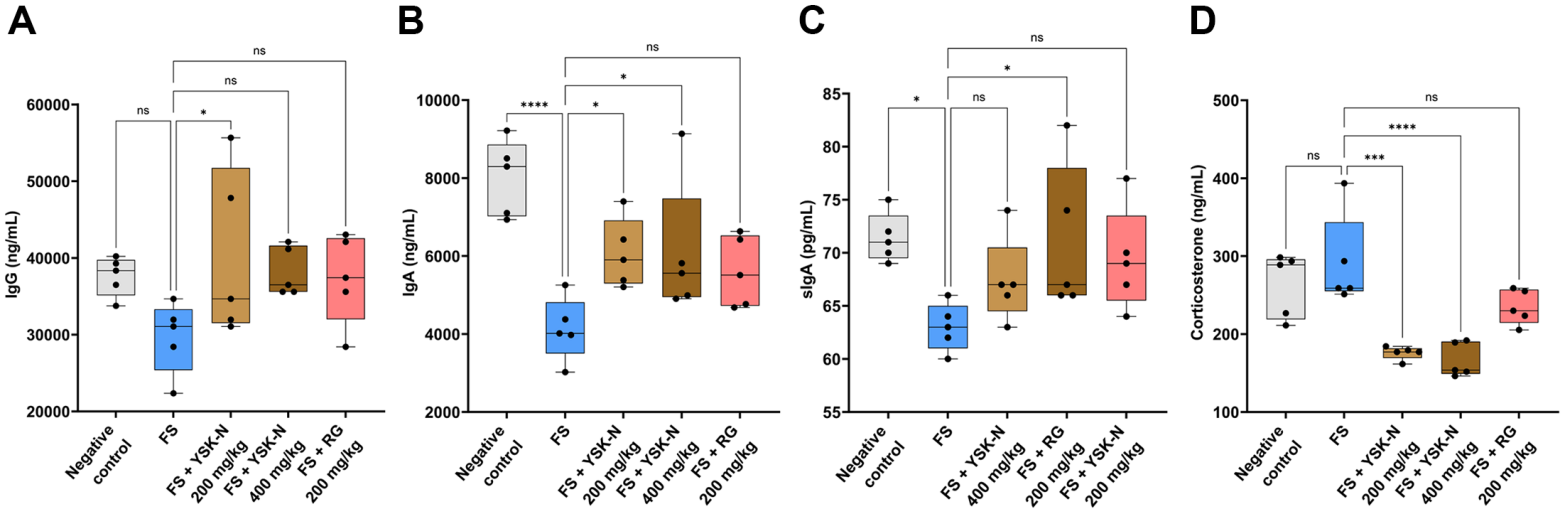
Effect of YSK-N on immunoglobulin and corticosterone production in the FS model. (**A-D**) Serum levels of IgG (**A**) IgA (**B**) sIgA (**C**) and corticosterone (**D**) were measured using ELISA (*n* = 5). Data were presented as mean ± SD and analyzed using one-way analysis of variance (**ANOVA**) (*p < 0.05, **p < 0.01, and ***p < 0.001). FS, forced swimming; YSK-N, *Nypa fruticans* Wurmb hot water extract; RG, red ginseng.

Immunoglobulins produced by B lymphocytes play essential roles in adaptive immunity [35]. IgG is most abundantly present in the blood (approximately 75%), followed by the lymph, cerebrospinal fluid, and peritoneal fluid, and it contributes to humoral immunity [36]. IgA and secretory IgA (sIgA) maintain mucosal homeostasis and serve as the primary barrier protection against pathogenic antigens and enteric toxins [37, 38]. Thus, we investigated whether YSK-N affects serum immunoglobulin production in an FS-induced immunosuppressed mouse model. Notably, serum IgG, IgA, and sIgA production was significantly decreased in the FS group compared to that in the negative control group (Fig. 5A-5C). However, YSK-N supplementation reversed this FS-induced decrease in serum immunoglobulin production (Fig. 5A-5C). Next, we evaluated the effects of YSK-N on serum corticosterone production in an FS-induced immune compromised mouse model. Corticosterone plays a critical role in immune function and stress response, and activation of the hypothalamic-pituitary-adrenal axis during stress leads to an increase in circulating corticosterone levels [39, 40]. As anticipated, serum corticosterone levels were elevated in the FS group compared to those in the negative control group (Fig. 5D). Notably, supplementation with YSK-N significantly reduced serum corticosterone production in a concentration-dependent manner compared to the FS group (Fig. 5D). Collectively, our findings revealed that YSK-N promoted serum immunoglobulin production and suppressed the production of the stress-induced hormone, corticosterone in an FS-induced immunosuppressed mouse model.

### YSK-N Elevated Immune Response- and NK Cell Function-Associated mRNA Expression in Splenocytes from FS Mouse Model

**Fig. 6:**
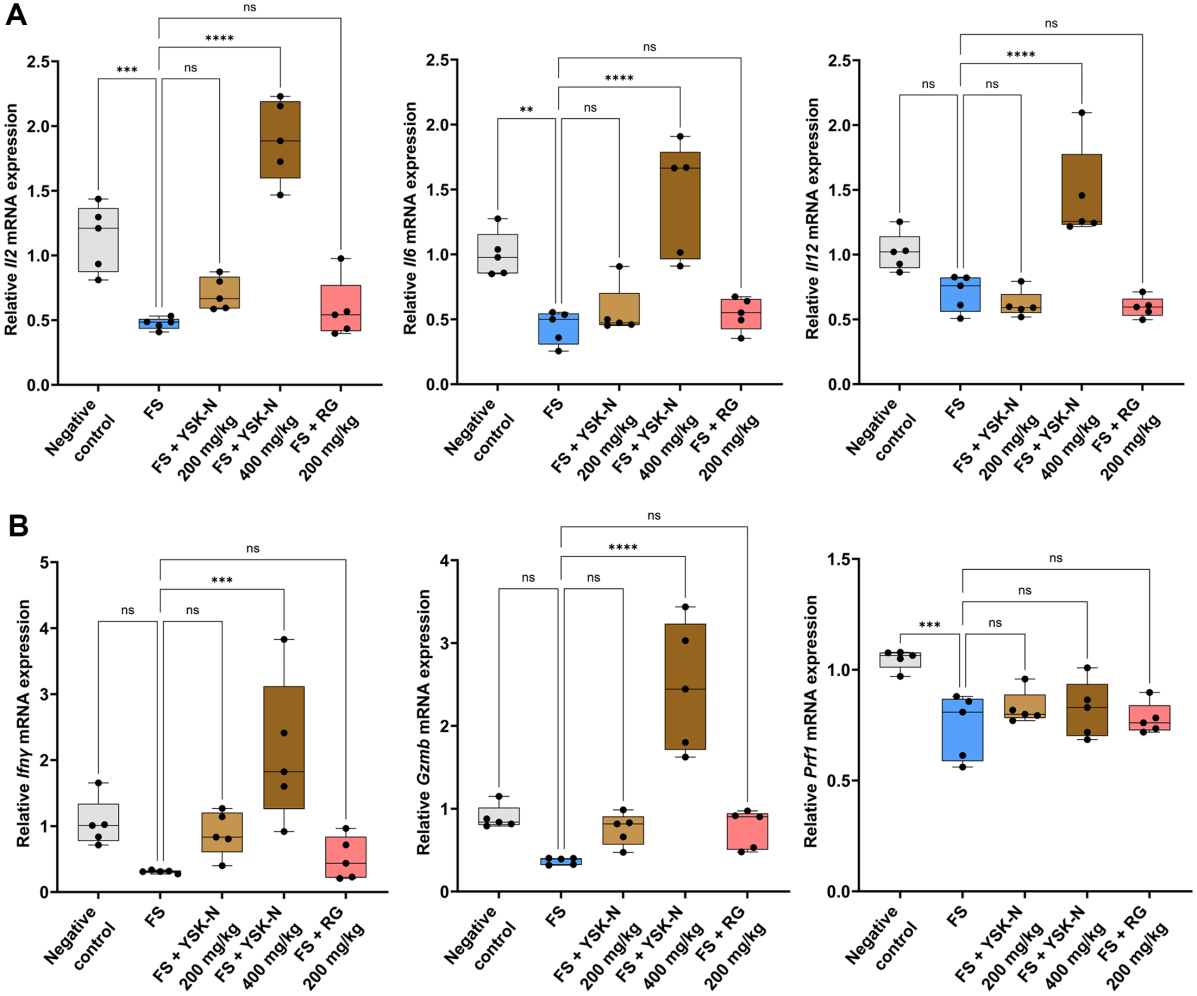
Effect of YSK-N on mRNA expression of genes related to immunostimulatory and NK cell functionality in the FS model. (**A-B**) Total RNA from the spleen of each mouse was utilized to quantify *Il2*, *Il6*, *Il12* (**A**), *Ifnγ*, *Gzmb*, and *Prf1* expression levels (**B**) using RT‐qPCR (*n* = 5). Data were presented as mean ± SD and analyzed using one-way analysis of variance (**ANOVA**) (*p < 0.05, **p < 0.01, and ***p < 0.001). FS, forced swimming; YSK-N, *Nypa fruticans* Wurmb hot water extract; RG, red ginseng.

To confirm that *ex vivo* immune response-related gene production, including IL2, IL6, and IL12p40, with ConA stimulation (Fig. 1B-1D) was reproduced in the FS mouse model, we used RT-qPCR to quantify *Il2*, *Il6*, and *Il12* mRNA expression in mouse splenocytes after FS. *Il2*, *Il6*, and *Il12* mRNA expression was lower in the FS group than in the negative control group (Fig. 6A). However, supplementation with YSK-N at 400 mg/kg revealed that significantly increased *Il2*, *Il6*, and *Il12* mRNA expression compared to that in the FS group (Fig. 6A). Next, we investigated whether YSK-N affected the expression of NK cell function-related genes in an FS-induced immune compromised model. As shown in Fig. 6B, *Ifnγ*, *Gzmb*, and *Prf1* mRNA expression was downregulated in the FS group in a manner similar to the results of splenic NK cell cytotoxicity analyses (Fig. 4D). Notably, oral administration of YSK-N elevated *Ifnγ*, *Gzmb*, and *Prf1* mRNA expression compared to levels in the FS group (Fig. 6B). Consequently, our studies showed that YSK-N increased *Il2*, *Il6*, *Il12*, *Ifnγ*, *Gzmb*, and *Prf1* mRNA expression in FS-induced immune compromised mouse model.

## Discussion

Our study showed that supplementation with YSK-N, an *N. fruticans* Wurmb hot water extract, recovered immune function in an FS-induced immunosuppressed mouse model. The major findings of our study are as follows: YSK-N promoted the proliferation and function of lymphocytes from draining and mesenteric lymph nodes without cytotoxicity in *ex vivo* experiments; YSK-N restored the FS-induced decrease in body and immune organ weight; YSK-N recovered the decreased population of lymphocytes and white blood cells in the whole blood under FS conditions; YSK-N improved the proliferation of splenocytes and increased splenic NK cell cytotoxicity in FS-induced immune compromised mouse model; YSK-N increased serum IgG, IgA, and sIgA production whereas decreased corticosterone production; and YSK-N increased *Il2*, *Il6*, *Il12*, *Ifnγ*, *Gzmb*, and *Prf1* mRNA expression in FS-induced immunosuppressed mouse model. Our observations collectively demonstrate that YSK-N recovered the suppressed immune status and functions induced by FS in a mouse model. Therefore, our findings suggested that YSK-N promotes immune function and has potential as an immunomodulatory functional material.

Irregular and intense exercise reduces the number of lymphocytes and decreases the proliferation of T and B lymphocytes [41-44]. Similarly, subjecting splenocytes isolated from mice to FS and stimulating them with ConA and LPS to assess T and B cell proliferation, the FS group exhibited significantly reduced lymphocyte proliferation [10]. Consistent with these findings, our studies demonstrated that the proliferative capacity of splenocytes in the presence or absence of ConA and LPS stimulation was significantly reduced in the FS group compared to that in the control group, and this was accompanied by a decrease in thymus and spleen weights. Studies have shown that as the impetus and intensity of exercise increase, the incidence of upper respiratory tract infections increases [45, 46]. Similarly, prolonged training or acute intense exercise reduces IgA production and the number and function of NK cells, which play critical roles in the initial immune response, thereby increasing the susceptibility to upper respiratory tract infections [47]. Furthermore, intensive exercise is associated with decreased levels of IgA, with reduced IgA levels reported in the saliva of swimmers. In swimmers, moderate-intensity acute exercise significantly reduces salivary IgA levels, whereas high-intensity chronic exercise lowers resting IgA levels and significantly elevates cortisol levels [48]. Similarly, in elite swimmers, salivary IgA levels decreased by 4.1% with each additional month of training and by 8.5% with each additional kilometer of training distance [49]. Consistent with these observations, our results revealed that the levels of serum IgG, IgA, and sIgA and the cytotoxicity of splenic NK cells were significantly decreased in the FS-induced immunosuppressed model, whereas the serum corticosterone level was increased.

These findings suggest that excessive exercise may impair immune homeostasis and inhibit the proliferation and activation of immune cells and mediators, thereby increasing susceptibility to infections and contributing to a higher disease incidence. In contrast, other studies demonstrated that regular, personalized scientific exercise regimens can enhance adaptive immune responses, including cell-mediated immune responses, in addition to humoral immune responses that promote antibody production [50-52]. Therefore, further multi-approach analyses, including epigenetic, metabolomic, and high-throughput analyses of biomarkers and related pathways across different individuals, sexes, and age groups, are needed to evaluate the immunomodulatory functions of exercise. Additionally, studies are needed to identify active substances in *N. fruticans* Wurmb extract using liquid chromatography-mass spectrometry and to elucidate the physiological activities and associated mechanisms of the identified substances.
